# Impaired Cellular Immunity and Cross‐Reactive Humoral Responses to Mpox Virus in HIV‐Infected Individuals Vaccinated with Vaccinia Virus Tiantan Strain

**DOI:** 10.1002/mco2.70399

**Published:** 2025-11-20

**Authors:** Qiao Zhang, Rui Song, Yu Huang, Meiyu Fang, Xiaoyou Chen, Danyang Li, Yanan Li, Xueqi Chi, Fengwen Xu, Jingchuan Zhong, Lan Chen, Zhixia Gu, Hongxin Zhao, Yuanyuan Zhang, Ning Han, Elie Antoun, Yanchun Peng, Tao Dong, Li Guo, Fei Guo, Lili Ren, Jianwei Wang, Ronghua Jin

**Affiliations:** ^1^ NHC Key Laboratory of Systems Biology of Pathogens and Christophe Mérieux Laboratory, National Institute of Pathogen Biology, Chinese Academy of Medical Sciences & Peking Union Medical College Beijing China; ^2^ Beijing Ditan Hospital Capital Medical University Beijing China; ^3^ Center For AIDS Research Chinese Academy of Medical Sciences & Peking Union Medical College Beijing China; ^4^ Chinese Academy of Medical Science (CAMS) Oxford Institute (COI), University of Oxford Oxford UK; ^5^ MRC Human Immunology Unit MRC Weatherall Institute of Molecular Medicine Radcliffe Department of Medicine University of Oxford Oxford UK; ^6^ Key Laboratory of Pathogen Infection Prevention and Control (Ministry of Education), State Key Laboratory of Respiratory Health and Multimorbidity, National Institute of Pathogen Biology, Chinese Academy of Medical Sciences & Peking Union Medical College Beijing China; ^7^ Key Laboratory of Respiratory Disease Pathogenomics, Chinese Academy of Medical Sciences & Peking Union Medical College Beijing China

**Keywords:** human immunodeficiency virus (HIV), humoral immune responses, memory T‐cell, monkeypox virus (MPXV), vaccinia virus

## Abstract

In the global response to the current mpox epidemic, understanding immune memory to the vaccinia virus and cross‐immunity to the mpox virus (MPXV) among people living with HIV (PLWH) is critical. Blood samples were collected from PLWH born between 1949 and 2002 without MPXV infection, as well as from sex‐ and age‐matched healthy donors (HD). Note that 62% and 56% of vaccinated PLWH born before 1980 had antibodies against the vaccinia virus Tiantan (VTT) strain and MPXV, respectively, though these seropositivity rates were lower than in HD (84% vs. 80%). Neutralizing antibodies were detected in 9% of PLWH, compared to 32% in HD. Notably, in PLWH with CD4 T‐cell counts below 500 cells/mm^3^, VTT‐IgG and MPXV‐IgG titers, as well as VTT‐specific memory B cells, were significantly reduced. In PLWH with CD4 T‐cell counts below 350 cells/mm^3^, CD4^+^ memory T‐cell responses were diminished, particularly in IFN‐γ and TNF‐α responses. In contrast, CD8^+^ T‐cell responses to MPXV were comparable in PLWH regardless of CD4 T‐cell counts. These findings highlight the diminished humoral and CD4^+^ T‐cell responses in PLWH, particularly in those with lower CD4 T‐cell counts, and underscore the necessity for targeted vaccination strategies in this population.

## Graphical abstract

This study examines MPXV immunity in PLWH, focusing on smallpox vaccine‐induced responses. PLWH with CD4⁺ T‐cell counts <500 cells/mm^3^ showed reduced VTT‐IgG, MPXV‐IgG, and NAbs, while CD4⁺ T‐cell responses were impaired at counts <350 cells/mm^3^. CD8⁺ T‐cell responses remained stable. Findings reveal diminished humoral and CD4⁺ T‐cell responses in immunocompromised PLWH, underscoring the need for tailored vaccination strategies.

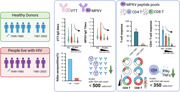

## 1 Introduction

Monkeypox (Mpox) is a viral disease caused by the monkeypox virus (MPXV), which belongs to orthopoxvirus [1]. Since the eradication of smallpox, MPXV has been the most concerning orthopoxvirus in humans in current years [2]. Studies have shown that smallpox vaccines provided approximately 85% cross‐protection against MPXV [3], suggesting that smallpox vaccine‐mediated immunity is effective against MPXV in persons born before 1980. However, the routine smallpox vaccination was terminated in most countries after smallpox eradication, resulting in a large proportion of the global population without immunity to orthopoxviruses. Moreover, antibody titers slowly wane over time in the smallpox‐vaccinated population, increasing the risk of MPXV infection [4, 5].

According to the report from the World Health Organization, approximately 52% of Mpox patients were living with HIV (PLWH). Patients who suffered MPXV and advanced HIV infection presented severe symptoms and longer disease courses. A cross‐sectional study according to GeoSentinel Network showed that patients with HIV were more likely to have diarrhea, perianal rash or lesions, and a higher rash burden [6]. Patients of HIV/MPXV coinfection had higher rates of bacterial superinfection [7]. As previously reported, all 27 deaths occurred in patients with suffered advanced HIV disease among 107 hospitalized mpox cases [8]. Thus, mpox rapidly escalated into a global epidemic in non‐endemic countries, disproportionately affecting PLWH.

Cross‐protective immunity has been demonstrated among orthopoxviruses, such as the vaccinia virus and MPXV. Note that 49 of 52 nonhuman primates (NHPs) vaccinated MVA‐BN survived under aerosol, intratracheal, or intravenous lethal challenge with MPXV clade I, whereas 0 of 35 unvaccinated NHPs survived. Human volunteers vaccinated with MVA‐BN developed cross‐reactive MPXV‐neutralizing antibodies and cell‐mediated responses [9]. Therefore, it is necessary to investigate the cross‐reactive immunity to MPXV induced by smallpox vaccination to collect more real‐world evidence.

The vaccinia virus Tiantan (VTT) strain was historically used to eradicate smallpox in China before 1980. Antibody and T‐cell responses induced by smallpox vaccination can persist for up to 75 years post‐vaccination in immunocompetent people [10]. A recent study showed that among healthy donors (HD) born before 1980 in China, 28.7%–60% had neutralizing antibodies, while at least 50% had memory T‐cell responses to VTT [11, 12]. However, PLWH had variable immune restoration following pathogen infections and vaccination, even though they were treated with antiretroviral therapy (ART) [13, 14]. The sustained immune responses induced by VTT and cross‐reactive immunity against MPXV are not well understood in PLWH.

In this study, we measured residual immunity of humoral and cellular immune responses to VTT and MPXV in PLWH to evaluate their infection risk and prognosis.

## 2 Results

### 2.1 Participants and Sample Collection

**FIGURE 1:**
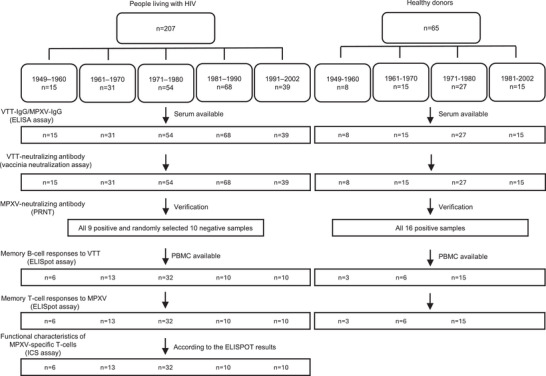
Flow chart of the study.

A total of 207 PLWH were recruited, among whom 100 (48.3%) were born between 1949 and 1980, and 107 (51.7%) were born between 1981 and 2002. The median age of the participants was 42 years (range 21–74; IQR 31–54), and 177 of 207 (85.5%) were male. Overall, there were 144 of 207 (69.6%) participants with CD4 T‐cell counts above 500 cells/mm^3^, 34 (16.4%) with CD4 T‐cell counts ranging from 350 to 499 cells/mm^3^, and 19 (9.2%) with CD4 T‐cell counts below 350 cells/mm^3^. HIV viral loads were undetectable in 140 (67.6%) participants, detectable but ≤1000 copies/mL in 58 (28.0%) participants, and >1000 copies/mL in nine (4.3%) participants. Note that 170 of 207 (82.1%) participants had been diagnosed with HIV infection for more than 3 years. All participants were compliant with regard to taking ART after being diagnosed with HIV infection (Table S1). Among the HD, eight were born during 1951–1960, 15 born during 1961–1970, 27 born during 1971–1980, and 15 born during 1981–2002. The flow diagram of the study is presented in Figure 1.

### 2.2 Antibody Responses Against VTT and Cross‐Reactivity With Mpox Virus

**FIGURE 2:**
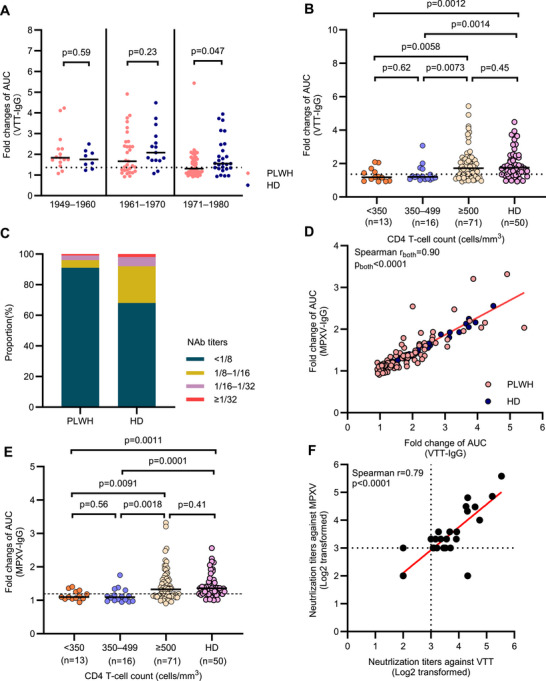
Antibody responses against VTT and cross‐reaction with MPXV among PLWH and matched HD. (A) VTT‐IgG titers in PLWH and healthy donors presented as fold changes of AUC by the year of birth. (B) VTT‐IgG titers presented as fold changes of AUC by CD4 T‐cell counts. (C) Prevalence of neutralizing antibody in PLWH and HD born before 1980. (D) The correlation between MPXV‐IgG titers and VTT‐IgG titers in PLWH and HD. (E) MPXV‐IgG titers in PLWH and HD presented as fold changes of AUC by CD4 T‐cell counts. (F) The correlation between the neutralizing antibody titers against VTT and the neutralizing antibody titers against MPXV. Comparisons between two independent groups were performed using the Mann–Whitney *U* test. Multiple comparisons of antibody titers were performed using the Kruskal–Wallis test followed by a post hoc Dunn's test. NAb titer was defined as 1/4 when it was below the limit of detection. The paired NAb titers against VTT and MPXV were compared using two‐tailed Wilcoxon matched‐pairs signed‐rank test. VTT, vaccinia virus Tiantan; MPXV, monkeypox virus; PLWH, people living with HIV; HD, healthy donor; AUC, area under the curve. The dotted lines indicate the detection limit of the assays.

Among the 100 PLWH born before 1980, 62 (62%) showed positive on VTT‐IgG, which was lower than that in matched HD (42/50, 84%, *p* = 0.0081; Figure S1). Two (1.9%) PLWH born after 1980 tested positive for VTT‐IgG, while no age‐matched HD showed positive (Figure S1). No significant differences in VTT‐IgG titers were observed between PLWH and HD born during 1949–1960 (*p* = 0.59) and 1961–1970 (*p* = 0.23), respectively. However, VTT‐IgG titers in PLWH born during 1971–1980 were lower than those of matched HD (*p* = 0.047; Figure 2A).

The PLWH with CD4 T‐cell counts below 350 (*p* = 0.014, *p* = 0.0058) and 350–499 cells/mm^3^ (*p* = 0.0014, *p* = 0.0073) showed lower VTT‐IgG positive rate and titers compared with PLWH with above 500 cells/mm^3^ and matched HD (PLWH with lower than 350 cells/mm^3^ vs. HD, *p* = 0.0008, *p* = 0.0012; PLWH with CD4 T‐cell counts 350–499 cells/mm^3^ vs. HD, *p* < 0.0001, *p* = 0.0014; Figure 2B, Figure S2). However, both the positive rate and levels of VTT‐IgG showed no difference between PLWH with above 500 cells/mm^3^ and matched HD (*p* = 0.16, *p* = 0.45; Figure 2B, Figure S2).

No detectable NAbs were found in serum from PLWH born during 1981–2002. The positive rate of NAbs against VTT was lower in PLWH compared with the matched HD born before 1980 (9% vs. 32%, *p* = 0.0008, Table S2, Figure 2C). Among the nine NAbs positive PLWH individuals, eight had CD4 T‐cell counts above 500 cells/mm^3^ and one had CD4 T‐cell counts 350–499 cells/mm^3^, with titers of 1/8–1/16 in five individuals (5.0%), 1/16–1/32 in three individuals (3.0%), and ≥1/32 in one individual (1.0%; Figure 2C).

The MPXV‐IgG titers were correlated with those of VTT‐IgG both in PLWH and HD (Spearman *r* = 0.90, *p* < 0.0001; Figure 2D). Most VTT‐IgG positive sera also tested positive for MPXV‐IgG in both PLWH (52/62, 83.9%) and HD (40/42, 95.2%; Figure S3). Similar to the VTT‐IgG, the overall seropositivity against MPXV in PLWH born before 1980 (56%) was lower than the matched HD (82%; *p* = 0.0019; Table S2). Higher seropositivity of MPXV‐IgG was found in PLWH born before 1970 than that born during 1971–1980 (*p* = 0.0002; Table S2). Compared to the matched HD, the titers of MPXV‐IgG were comparable in PLWH born before 1970 (*p* = 0.86) but lower in PLWH born during 1971–1980 (*p* = 0.0026; Figure S4). In PLWH born before 1980 with different CD4 T‐cell counts, the MPXV‐IgG positivity rates and titers followed similar trends to VTT‐IgG (Figure 2E, Figure S5). Meanwhile, different ART regimens did not result in significant differences in VTT‐IgG titers, MPXV‐IgG titers, or seropositivity rates (all *p* > 0.05, Figure S6).

A total of 25 VTT‐NAbs positive serum samples were used to evaluate the cross‐reactivity on MPXV, including nine from PLWH and 16 from the matched HD. All sera were positive on MPXV‐NAbs with titers ranging from 1/8 to 1/48. The NAb titers against MPXV were correlated with NAb titers against VTT (Spearman *r* = 0.79, *p* < 0.0001; Figure 2F).

These findings indicated the presence of cross‐reactive NAb against MPXV induced by VTT. However, the lower positive rate and titers of IgG and NAbs against VTT and MPXV in PLWH with CD4 T‐cell counts below 500 cells/mm^3^ suggested the impaired humoral responses.

### 2.3 Memory B‐Cell Responses Against VTT

**FIGURE 3:**
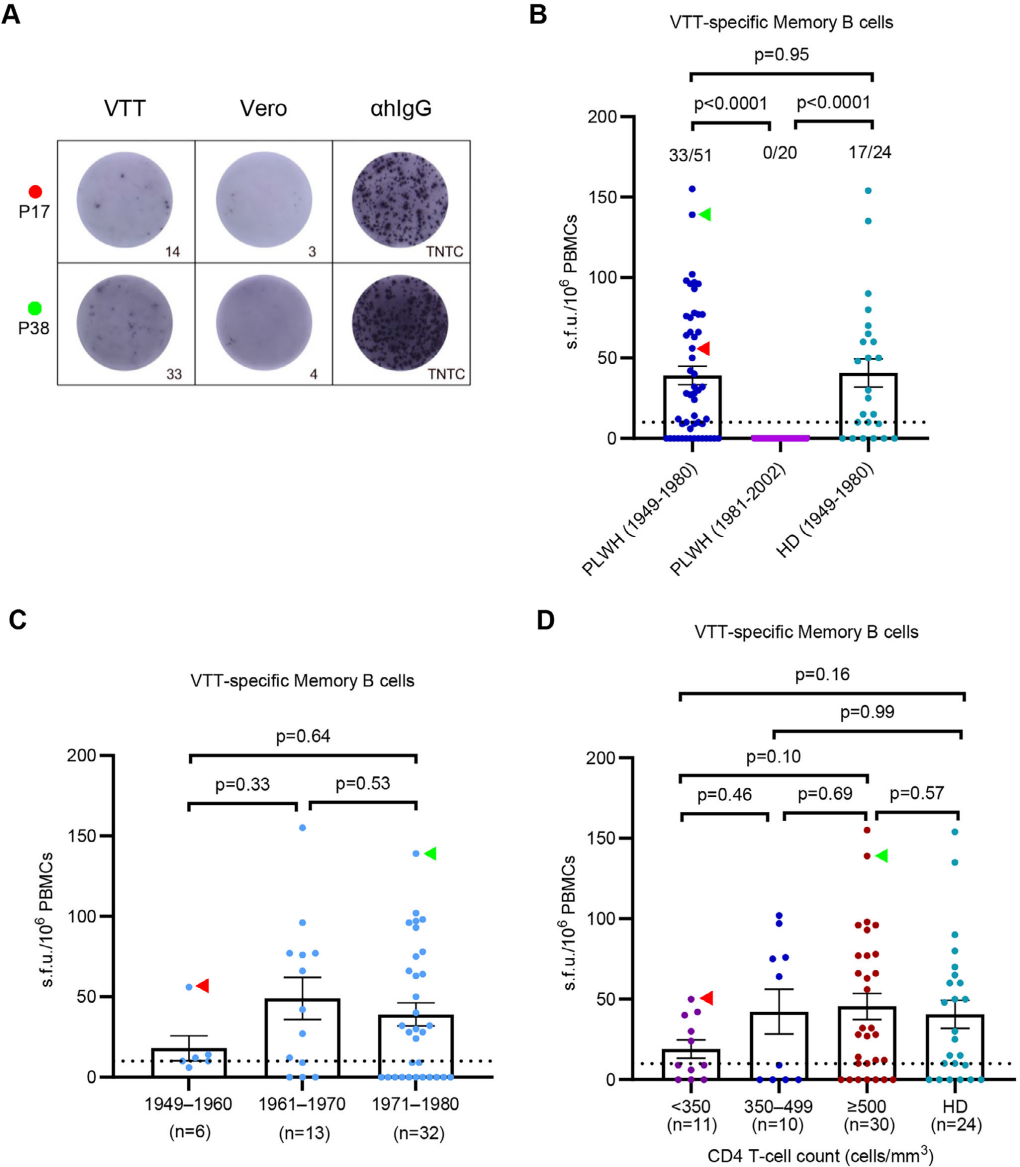
Memory B‐cell responses to VTT measured by IgG ELISpot according to year of birth and CD4 T‐cell counts. (A) Two representative samples of the IgG ELISpot responses against VTT. Vero cells were used as negative control, and anti‐human IgG mAb (MT91/145) was used as positive control. Individuals P17 and P38 in red and green dot and arrowheads, respectively. (B) Magnitude of VTT‐specific memory B‐cell responses in PLWH born during 1949–1980 (*n* = 51) and during 1981–2002 (*n* = 20), and in healthy donors born during 1949–1980 (*n* = 24). (C) Magnitude of VTT‐specific memory B‐cell responses by birth cohort in 51 PLWH born during 1949–1980. (D) Magnitude of VTT‐specific memory B‐cell responses by CD4 T‐cell counts in 51 PLWH and 24 healthy donors born during 1949–1980. Multiple comparisons of memory B‐cell responses were performed using the Kruskal–Wallis test followed by a post hoc Dunn's test. αhIgG, anti‐human IgG mAb; TNTC, too numerous to count. The dotted lines indicate the detection limit of the assays. Data are presented as the mean and standard error of mean (SEM).

Peripheral blood mononuclear cell (PBMC) samples from 71 PLWH were utilized to evaluate memory B‐cell responses against vaccinia virus. Among the participants, 51 were born between 1949 and 1980, and 20 were born between 1981 and 2002. The magnitude of VTT‐specific memory B‐cell responses was measured using an ex vivo antigen‐specific IgG ELISpot assay (Figures 1 and 3A). VTT‐specific memory B‐cell responses were detected in 33 out of 51 PLWH born before 1980 (64.7%) and in 17 out of 24 matched HD born before 1980 (70.8%, *p* = 0.98; Figure 3B). However, no VTT‐specific memory B‐cell responses were observed in PLWH born after 1980. No significant correlation was found between the magnitude of memory B‐cell responses to VTT and the year of birth (all *p* > 0.05, Figure 3C). Meanwhile, no significant correlations were observed between VTT‐specific memory B‐cell magnitude and VTT IgG or neutralizing antibody titers (Figure S7). However, the positive rate of VTT‐specific memory B‐cell responses in PLWH with CD4 T‐cell counts below 500 cells/mm^3^ was lower compared with those with above 500 cells/mm^3^ (47.6% vs. 76.7%, *p* = 0.033, Table S3), although the magnitude of VTT‐specific memory B‐cell responses among varying CD4 T‐cell counts was not significantly different (all *p* > 0.05, Figure 3D).

These findings collectively indicated the presence of VTT‐specific memory B‐cell in PLWH who had previously received smallpox vaccine. However, the lower positive rate of memory B‐cell against VTT in PLWH with CD4 T‐cell counts below 500 cells/mm^3^ diminishes VTT‐elicited humoral immunity.

### 2.4 Cross‐Reactive Memory T‐Cell Responses Against Mpox Virus

**FIGURE 4:**
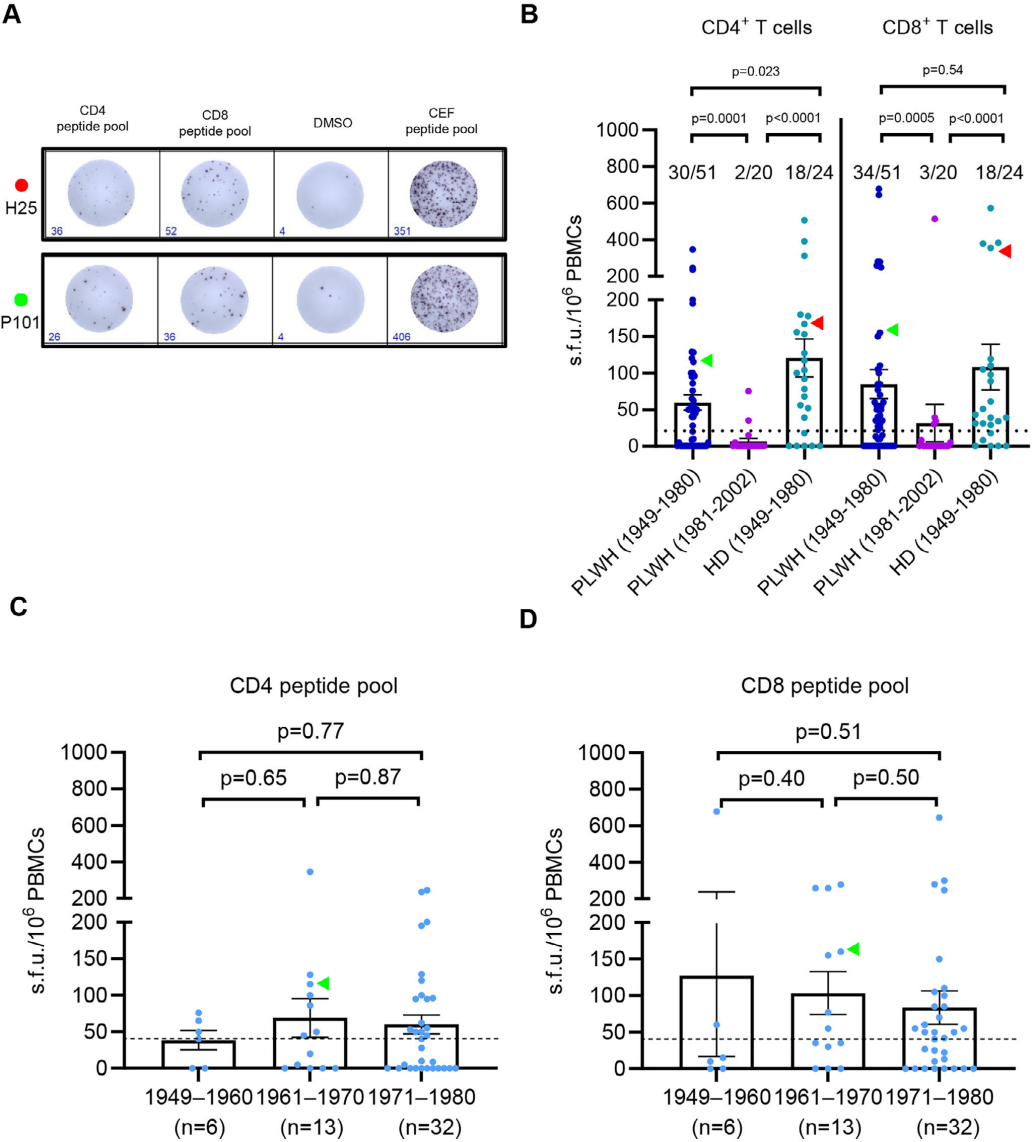
Memory T‐cell responses to MPXV measured by IFN‐γ ELISpot according to year of birth. (A) Two representative samples of the IFN‐γ ELISpot responses against MPXV peptide pools. DMSO was used as a negative control, and the pool containing CMV‐, EBV‐, and IFV‐specific epitopes (CEF peptide pool) was used as a positive control. Individuals H25 and P101 in red and green dot and arrowheads, respectively. (B) Magnitude of T‐cell IFN‐γ responses against CD4 and CD8 peptide pools in PLWH born during 1949–1980 (*n* = 51) and during 1981–2002 (*n* = 20), and in healthy donors born during 1949–1980 (*n* = 24). Magnitude of IFN‐γ T‐cell responses against MPXV CD4 peptide pool (C); CD8 peptide pool (D) by birth cohort in 51 PLWH born during 1949–1980. Multiple comparisons of memory T‐cell responses were performed using the Kruskal–Wallis test followed by a post hoc Dunn's test. CMV, cytomegalovirus; EBV, Epstein‐Barr virus; IFV, Influenza viruses. The dotted lines indicate the detection limit of the assays. Data are presented as mean and SEM.

To evaluate the cross‐reactive memory T‐cell responses against MPXV in PLWH, we conducted an ex vivo interferon‐γ (IFN‐γ) ELISpot assay to measure the magnitude of T‐cell responses, using CD4 and CD8 peptide pools (Figures 1 and 4A). IFN‐γ T‐cell responses against the CD4 MPXV peptide pools were detected in 30 of 51 (58.8%) of PLWH and 18 of 24 (75%; *p* = 0.21) of matched HD born before 1980. Only two out of 20 PLWH born after 1980 exhibited positive IFN‐γ T‐cell responses (Figure 4B). Furthermore, positive IFN‐γ T‐cell responses against the CD8 MPXV peptide pools were found in 34 of 51 (66.7%) of PLWH and 18 of 24 (75%; p = 0.59) of matched HD born before 1980, and three PLWH born after 1980 exhibited positive (Figure 4B). No significant difference was found between the magnitude of cross‐reactive memory T‐cell responses against MPXV and year of birth (all *p* > 0.05; Figure 4C,D).

**FIGURE 5:**
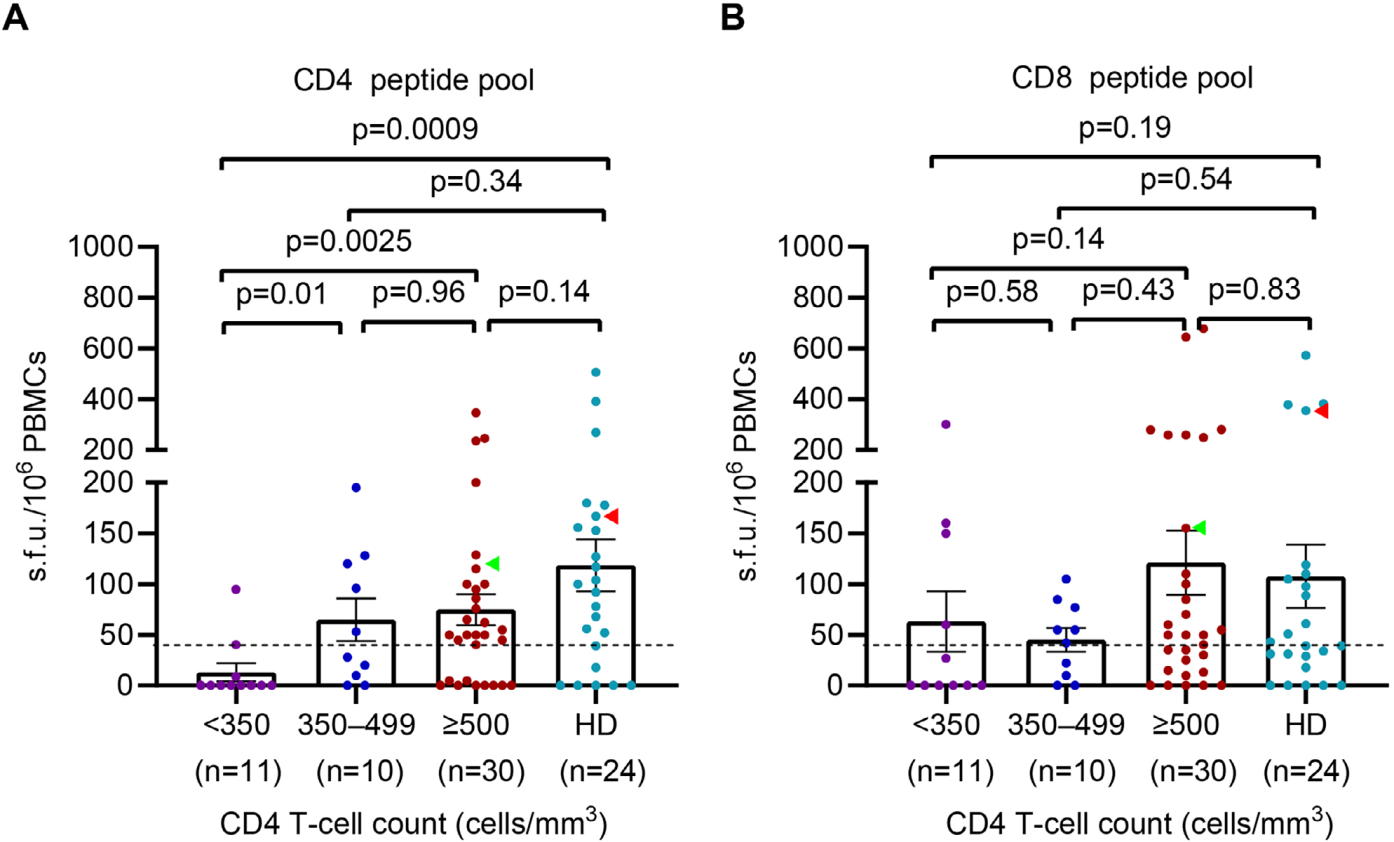
Memory T‐cell responses to MPXV peptide pools measured by IFN‐γ ELISpot according to CD4 T‐cell counts. (A) Magnitude of IFN‐γ T‐cell responses against MPXV CD4 peptide pool by CD4 T‐cell counts in 51 PLWH and 24 healthy donors born during 1949–1980. (B) Magnitude of IFN‐γ T‐cell responses against MPXV CD8 peptide pool by CD4 T‐cell counts in 51 PLWH and 24 healthy donors born during 1949–1980. Multiple comparisons of memory T‐cell responses were performed using the Kruskal–Wallis test followed by a post hoc Dunn's test. Individuals H25 and P101 in red and green dots and arrowheads, respectively. The dotted lines indicate the detection limit of the assays. Data are presented as the mean and SEM.

The magnitude of IFN‐γ T‐cell responses to the CD4 peptide pool was lower in PLWH compared to matched HD (*p* = 0.023, Figure 4B), while no significant difference was observed in responses to the CD8 peptide pool between the PLWH and HD (*p* = 0.54, Figure 4B). As expected, the magnitude of T‐cell responses against MPXV peptides was contracted in PLWH with CD4 T‐cell counts below 350 cells/mm^3^ compared with those PLWH with 350–499 cells/mm^3^ (*p* = 0.010), 500 cells/mm^3^ (*p* = 0.0025), and matched HD (*p* = 0.0009; Figure 5A). There was no significant difference in the magnitude of T‐cell response against CD4 peptide pool among PLWH with CD4 T‐cell counts of 350–499 cells/mm^3^, more than 500 cells/mm^3^ (all *p* > 0.05, Figure 5A) compared to HD. The positive rate of IFN‐γ T‐cell responses against the CD4 peptide pool in PLWH with CD4 T‐cell counts below 500 cells/mm^3^ was lower compared with those with more than 500 cells/mm^3^ (33.3% vs 70.0%, *p* = 0.0096, Table S3). The magnitude of IFN‐γ T‐cell responses to the CD8 peptide pool showed no difference between PLWH with varying CD4 T‐cell counts and matched HD (all *p* > 0.05, Figure 5B). Similarly, the positive rate of IFN‐γ T‐cell responses to the CD8 peptide pool showed no difference between PLWH with different CD4 T‐cell counts (47.6% vs. 70.0%, *p* = 0.11, Table S3).

Collectively, these findings suggest that memory CD8^+^ T‐cell responses against MPXV in PLWH are unrestricted by variations in CD4 T‐cell counts. However, low CD4 T‐cell counts diminished cross‐reactive memory CD4^+^ T‐cell function to MPXV in PLWH who had previously received smallpox vaccine.

### 2.5 Functional Characteristics of Cross‐Reactive Memory T‐Cell Responses Against Mpox Virus in PLWH

**FIGURE 6:**
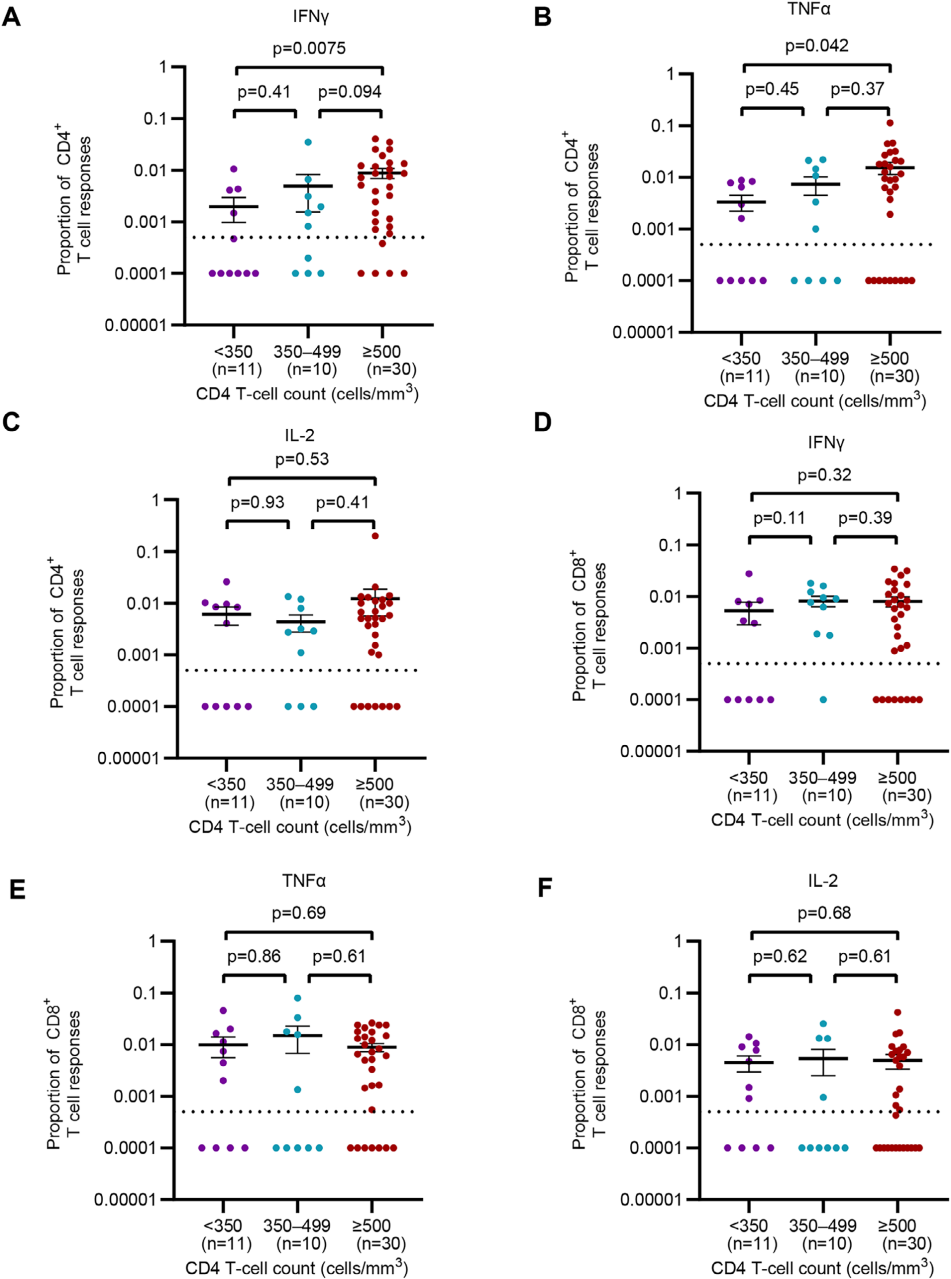
Functional characterization of MPXV‐reactive T‐cells in people living with HIV. Cytokine‐producing T cells were detected by ICS after incubation with MPXV peptide pools in 51 PLWH born before 1980. Comparison of the relative proportion of MPXV CD4 peptide pool‐reactive IFN‐γ (A), TNF‐α (B), and IL‐2 (C) T cells among PLWH with different CD4 T‐cell counts. Comparison of the relative proportion of MPXV CD8 peptide pool‐reactive IFN‐γ (D), TNF‐α (E), and IL‐2 (F) T cells among PLWH with different CD4 T‐cell counts. Multiple comparisons of cytokine generation of memory T cell were performed using the Kruskal–Wallis test followed by a post hoc Dunn's test. ICS, intracellular cytokine staining; IFN‐γ, interferon γ; TNF‐α, tumor necrosis factor α; IL‐2, interleukin 2. Data are presented as the mean and SEM.

To further assess the functional cross‐reactive memory T‐cell responses against MPXV in PLWH, we performed an intracellular cytokine staining (ICS) assay in PLWH born before 1980 (Figure S6). The proportions of both IFN‐γ (*p* = 0.0075) and TNF‐α (*p* = 0.042) responses to the CD4 peptide pool were lower in PLWH with CD4 T‐cell counts below 350 cells/mm^3^ compared to those with counts above 500 cells/mm^3^ (Figure 6A,B). However, the proportions of IL‐2 responses to the CD4 peptide pool, as well as IFN‐γ, TNF‐α, and IL‐2 responses to the CD8 peptide pool (Figure 6C–F) showed no differences among PLWH with varying CD4 T‐cell counts (all *p* > 0.05). Additionally, the positive rate of at least one cytokine response against the CD4 peptide pool in PLWH with CD4 T‐cell counts below 500 cells/mm^3^ was lower than those with higher CD4 T‐cell counts (85.7% vs. 100.0%, *p* = 0.033, Table S3). In contrast, no significant difference in the positive rate of cytokine responses to the CD8 peptide pool was observed between PLWH with different CD4 T‐cell counts (90.5% vs. 96.7%, *p* = 0.36, Table S3). These findings suggested that PLWH with CD4 T‐cell counts below 500 cells/mm^3^ should be considered intensively due to the deficiency of memory CD4^+^ T‐cell response when they were infected by MPXV.

## 3 Discussion

In this study, we found that 62% of PLWH born during 1949–1980 were seropositive on VTT‐IgG, 56% were positive on MPXV‐IgG. The NAb titers against MPXV were correlated with those against VTT. PLWH with CD4 T‐cell counts less than 500 cells/mm^3^ exhibited lower MPXV‐IgG titers and lower positive rate of VTT‐specific memory B‐cell responses. Among PLWH born during 1949–1980, only 9% had detectable, low‐level NAb responses against VTT, which was lower than in matched HD, despite comparable VTT‐specific memory B‐cell responses between PLWH and HD. Additionally, among all PLWH born during 1949–1980, 58.8% had CD4^+^ and 66.7% had CD8^+^ T‐cell responses against MPXV. The magnitude and proportion of T‐cell responses against the CD4 peptide pool were reduced in PLWH with CD4 T‐cell counts below 500 cells/mm^3^, with lower IFN‐γ and TNF‐α responses in CD4^+^ T MPXV memory T cell. In contrast, CD8^+^ T‐cell responses against MPXV were comparable in PLWH, regardless of the CD4 T‐cell counts. To our knowledge, this is the first study of VTT and MPXV immunity in the Chinese HIV‐infected population, investigating both the humoral and cellular immune responses.

About 56% of PLWH born before 1980 had cross‐reactive MPXV‐IgG in this study, including the oldest individual aged 74 years. Our results are consistent with a cross‐sectional study showing that serum antibody can persist for up to 75 years post vaccination [10], as well as the data from additional studies [15, 16]. However, we observed a low prevalence of NAbs against VTT in HIV‐infected individuals born before 1980, which is consistent with previous reports conducted in the Chinese population [11, 17]. In contrast, a study from other countries showed that 50% of individuals who had received vaccinia virus vaccines 20 years earlier had vaccinia virus NAb titers above 1/32 [10]. This discrepancy may be attributed to a gradual decline of immune function, as well as immune dysfunction in HIV‐infected individuals, as well as the different evaluation methods. Regardless of these differences, protective immunity against lethal smallpox virus infection can persist for a lifetime without re‐exposure [18]. The proteins of VACV have high sequence homology with those of MPXV. Thus, VTT‐elicited antibodies have significant cross‐protective activity against MPXV.

Following smallpox vaccination, memory B cells against vaccinia virus can persist for up to 30 years [19]. We observed that 64.7% of PLWH born before 1980 exhibited VTT‐specific memory B‐cell responses, comparable to vaccinated healthy individuals. However, consistent with previous studies, there was no significant correlation between the frequency of VTT‐specific memory B cells and serum antibody titers [11, 19, 20]. This suggests that in maintaining long‐term humoral immunity against vaccinia virus, serum antibody levels and memory B cells are equally stable but independently maintained, without a direct cause‐and‐effect relationship. Studies have shown that HIV‐infected individuals experience memory B‐cell loss and functional decline [21, 22]. Influenza‐specific memory B‐cell responses were significantly lower in HIV‐infected than in HIV‐negative individuals and were directly correlated with CD4 T‐cell counts [23]. Similarly, we observed a lower positivity rate of VTT‐specific memory B‐cell responses in PLWH with low CD4 cell counts. This further explains that the maintenance of B‐cell memory responses in HIV‐infected individuals is dependent on CD4 T cells.

CD4^+^ T‐cell and CD8^+^ T‐cell immune responses are mounted after smallpox vaccination [11, 24] and MPXV infection [25]. However, data on cross‐reactive T‐cell responses against MPXV in the HIV‐infected population have been lacking. We observed that 58.8% of PLWH had CD4^+^ T‐cell responses and 66.7% of PLWH had CD8^+^ T‐cell responses to MPXV among PLWH born before 1980. This positive rate is similar to that of our previous report showing about 65.7% of healthy individuals could maintain T‐cell memory responses after VTT vaccination [11]. These data also suggest that the T‐cell immunity against smallpox can be maintained for decades after vaccination in the absence of antigen re‐exposure in PLWH. T‐cell‐mediated immunity has been shown to confer protection against vaccinia virus infection in B‐cell‐deficient or MHC II‐deficient mice [26, 27].

Immune restoration disorder occurs in about 40% of severe HIV patients who commence ART. The risk of immunopathology increases due to inadequate control of HIV infection [28]. Immunocompromised persons, including HIV‐infected individuals, are at increased risk of severe outcomes [7]. It has been reported that advanced HIV infection presents a high prevalence of fulminant dermatological and systemic manifestations, and death in people with Mpox [8]. Cases of Mpox patients living with HIV and had high CD4 T‐cell counts had similar outcomes compared to HIV‐negative individuals [29, 30]. However, Mpox patients living with HIV and had low CD4 T‐cell counts had worse clinical outcomes and higher mortality [8]. Two patients with Mpox and AIDS (CD4 T‐cell counts less than 100 cells/mm^3^) died due to systemic complications [31]. Thus, PLWH with low CD4 T‐cell counts are at high risk of MPXV infection. MVA‐BN vaccine effectively prevents lethal monkeypox disease [32]. Following vaccination, the magnitude and kinetics of the immune response are comparable in the short term between HIV‐infected individuals (with CD4⁺ T‐cell counts >250 cells/mm^3^) and non‐infected groups. However, further evaluation of vaccine efficacy is required [33].

Follicular helper T cells, a subtype of CD4 T cells, are essential in facilitating germinal center (GC) formation and differentiating GC B cells into memory B and plasma cells [34]. In this study, we observed that the positive rates of NAbs against VTT in PLWH and the CD4^+^ T‐cell responses against MPXV were lower than those in the matched HD. The positive rate of IgG, memory B‐cell responses, and CD4^+^ T‐cell responses against VTT and MPXV were lower in PLWH with CD4 T‐cell counts below 500 cells/mm^3^, compared with the PLWH with CD4 T‐cell counts above 500 cells/mm^3^. These data suggest that both HIV status and CD4 T‐cell counts reduce the durability of vaccine‐mediated immunity and determine the levels of cross‐reactive adaptive immunity against MPXV in PLWH. Adjusting vaccination strategies is expected to enhance vaccine effectiveness. (1) Extending the MVA‐BN dosing interval: In a two‐dose regimen, a 28‐day interval yields a long‐term protective efficacy of 66.6% (CI: 48.8−78.2). With the second dose delayed to 730 days, the predicted efficacy at 10 years post‐booster increases to 77.6% (CI: 65.7–85.4) [35]. (2) Stratified immunization for high‐risk groups: For PLWH with CD4 T‐cell counts between 350 and 500 cells/mm^3^, a booster strategy is recommended. For PLWH with counts below 350, priority should be given to passive immunization agents (e.g., neutralizing antibodies such as A138 and B026 [36]).

Our study has several limitations. First, the sample sizes are relatively small, and PBMC samples were not available for some patients to investigate the B‐ and T‐cell response. Our findings should be further validated with a larger PLWH cohort, which could improve the impact of our results. Second, we only confirmed the history of smallpox vaccination through participants' self‐reports and vaccination scars, without obtaining the exact timing of vaccination. This resulted in a lack of analysis on the dynamics of residual immune responses to smallpox vaccines in the study.

In summary, we have evaluated residual immune responses to smallpox vaccines and cross‐reactive immunity to MPXV in PLWH. Our study shows that those with low CD4 T‐cell counts had low MPXV‐IgG titers and low VTT‐specific memory B cells. Most PLWH had no or low NAb titers against VTT and MPXV. Over 58% PLWH born before 1980 had cross‐reactive memory T‐cell responses to MPXV. The magnitude of T‐cell responses against the CD4 peptide pool in PLWH with CD4 T‐cell counts below 500 cells/mm^3^ was lower than those in PLWH with high CD4 T‐cell counts. The low levels of cross‐reactive antibody and memory T‐cell responses against MPXV and decreased durability of vaccine‐mediated immune response in PLWH with low CD4 T‐cell counts highlight the necessity of MPXV vaccine in the high‐risk population and aim to induce long‐term protective effective humoral and cellular immunity against MPXV.

## 4 Materials and Methods

### 4.1 Study Design and Participants

PLWH was recruited from Red Ribbon Center in Beijing Ditan Hospital Capital Medical University (Beijing, China) between July 2 and July 9, 2023. The HD participated in routine health assessments were recruited from the hospital as 2:1 matched by age and sex with PLWH bore at 1949–1980. HD bore after 1980 was also recruited. Participants over 18 years of age were eligible. Vaccination status verification was conducted through dual methods: examination of self‐reported accounts and clinical observation of distinctive petaloid scarring patterns on the deltoid area. The ART regimens were derived from electronic medical records within a 1‐month timeframe. Non‐anticoagulant and anticoagulant blood samples were collected from participants. Following centrifugation, separated serum aliquots underwent cryopreservation at −80°C prior to batch testing. A dual‐label anonymization system was systematically implemented across all biospecimens during pre‐analytical processing. The study was approved by the Institutional Review Boards of Beijing Ditan Hospital Capital Medical University (2023‐025). The written informed consent was obtained from all subjects before inclusion. Detailed information of the subjects could be found in the demographic characteristics of patients in this study (Table S1).

### 4.2 Serum and PBMC Isolation

Venous blood was collected through venipuncture into serum separator tubes. Serum samples were stored at −80°C until testing. Venous blood specimens anticoagulated with EDTA (ethylenediaminetetraacetic acid) were acquired through veno venous puncture using standard collection protocols. The separation of peripheral blood mononuclear cells (PBMCs) was conducted utilizing density‐based centrifugation methodology with commercially available SepMate tubes (STEMCELL Technologies, Cambridge, MA) following manufacturer's instructions. Isolated PBMCs were cryopreserved in liquid nitrogen before analysis.

### 4.3 Cells and Viruses

Vero cell lines (ATCC CCL‐81) were cultured using Dulbecco's modified Eagle medium (DMEM, Gibco, Grand Island, NY) containing 10% (v/v) heat‐inactivated fetal bovine serum (FBS, Gibco). Cell culture maintenance involved incubation under conditions of 37°C with 5% CO_2_. Cells were routinely tested negative for mycoplasma. A modified VTT strain carrying a Gaussia luciferase gene was constructed as previously reported [11]. An MPXV strain (IPBCAMS‐MP05‐1/2023) was isolated from MPXV patient lesion sample in biosafety level 3 laboratory by National Institute of Pathogen Biology, Chinese Academy of Medical Sciences.

### 4.4 Enzyme‐Linked Immunosorbent Assay (ELISA)

Background signal subtraction was performed to determine immunoglobulin (Ig) G titers. Curve analysis was conducted using GraphPad Prism 10.1 (GraphPad Software, San Diego, CA) to quantify the area under the curve (AUC) values. Uninfected Vero cell lysates served as negative controls in all serum sample analyses. Prior to subsequent analysis, background correction was applied to quantify both VTT‐specific and MPXV‐specific IgG titers. ELISA threshold determination was based on the computation of AUC ratios between VTT/Vero and MPXV/Vero. Seropositivity was defined as VTT/Vero AUC ratios ≥1.3 and MPXV/Vero AUC ratios ≥1.2 for their respective IgG titers.

### 4.5 Microneutralization Assay Against VTT

Serum neutralization titers were assessed through the Gaussia luciferase reporter system in vaccinia virus neutralization experiments. Serum specimens underwent serial twofold dilution (initial dilution 1:4) and were co‐incubated with equivalent volumes of rTV‐GLuc reporter virus (1000 PFU) at 37°C for 60 min. The virus‐serum complexes were subsequently transferred to Vero E6 cells (ATCC CCL‐81) for incubating 60 minutes. Following PBS washing, cells were maintained in 200 µL DMEM supplemented with 2% FBS. Post 24‐h incubation, 40 µL aliquots of cell culture supernatant were collected and combined with 60 µL coelenterazine substrate (Promega, Madison, WI, USA). Luminescent signals were quantified utilizing the Steady‐Glo Luciferase Detection System (Promega). Neutralization titers (NT50) were determined as the reciprocal of the maximum serum dilution achieving 50% reduction in luminescence intensity compared to virus‐free controls.

### 4.6 Plaque Reduction Neutralization Test (PRNT)

Serum samples underwent serial twofold dilutions (starting at 1:4) and were pre‐incubated with MPXV (75 PFU, as quantified in Vero cells, ATCC CCL‐81). The virus was isolated from Mpox patient lesions in a BSL‐3 laboratory. Following 1 h of incubation at 37°C, the virus‐serum mixtures were transferred to Vero cells cultured in 12‐well plates (Costar). After 2 h, the mixtures were aspirated and replaced with 1 mL of fresh overlay medium (DMEM supplemented with 2% FBS and 1% methylcellulose). Three days post‐infection, plaques were visualized by crystal violet staining and enumerated manually. Each serum dilution was tested in duplicate. Neutralizing antibody titers (PRNT50) were determined as the maximal serum dilution yielding >50% plaque reduction.

### 4.7 Memory B Cell ELISpot Assays

Memory B‐cell responses were evaluated by the ELISpot Flex: Human IgG (ALP) kit (Mabtech, Sweden) following the manufacturer's instructions. The ELISpot plates were pre‐coated with 1 × 10⁷ PFU of heat‐inactivated purified vaccinia virus. Lytic Vero cells served as negative controls in each assay. The anti‐human IgG monoclonal antibody MT91/145 was employed as a positive control. Following 48‐h pre‐stimulation with a mixture of R848 (1 µg/mL) and rhIL‐2 (10 ng/mL), 2 × 10⁵ PBMCs were seeded onto the plates. The plates were incubated at 37°C for 24 h. Post cell removal, the plates underwent washing. A detection antibody diluted in 0.5% FBS with PBS was applied and incubated for 1 h at room temperature. Following washing, streptavidin‐ALP was introduced and incubated for 1 h at room temperature. Substrate solutions were subsequently added, and development continued until distinct spots appeared. Color development was terminated via tap water washing. Spots were quantified using an AID ELISPOT reader system (AID Diagnostika GmbH, Strassberg, Germany). To determine VTT‐specific memory B‐cell responses, the mean spot count from negative control wells was subtracted from test wells. Results were expressed as spot‐forming units per 10⁶ PBMCs (s.f.u./10^6^ PBMCs). A response was deemed positive if the mean spot count exceeded both twofold the negative control and 10 s.f.u./10⁶ PBMCs.

### 4.8 Peptide Synthesis

Two peptide pools were synthesized by ProImmune (London, UK) with >90% purity: (1) a CD4^+^ T‐cell epitope pool containing 248 MPXV‐specific 9–23mer peptides, and (2) a CD8^+^ T‐cell epitope pool containing 276 MPXV‐targeting 9–16mer peptides. The peptides were derived from conserved regions across viral life cycle proteins (immediate early, early, intermediate, and late phases) of both VACV and MPXV. A control CEF peptide pool (>90% purity, SciLight Biotechnology Co., Beijing, China) containing immunodominant epitopes from CMV, EBV, and IFV was included for comparative analysis.

### 4.9 Ex Vivo IFN‐γ ELISpot Assays

Ex vivo IFN‐γ ELISpot assays (Human IFN‐γ ELISpot kit, Mabtech, Sweden) were performed to assess memory T‐cell responses according to the manufacturer's protocol. PBMCs (2 × 10⁵ cells/well) were incubated for 24 h at 37°C with MPXV‐specific CD4 or CD8 peptide pools. Negative and positive controls included DMSO and CEF peptide pool, respectively. Spot enumeration was conducted with an AID ELISPOT Reader System. MPXV‐specific responses were calculated by subtracting the mean negative control spots and expressed as spot‐forming units per 10⁶ PBMCs (s.f.u./10⁶ PBMCs). A positive response was defined as a greater than or equal to twofold increase over the negative control and ≥20 s.f.u./10⁶ PBMCs.

### 4.10 Intracellular Cytokine Staining

PBMCs were stimulated with peptide pools (10 µg/mL) for 1 h, followed by treatment with brefeldin A (GolgiPlug, Biolegend, San Diego, CA) and monensin (GolgiStop, Biolegend) for 5 h. Cell viability was assessed using BD Horizon Fixable Viability Stain 510 (BD Biosciences, NJ, CA). Surface markers were stained with PerCP‐Cy5.5‐anti‐CD3, BV650‐anti‐CD4, and PE‐Cy7‐anti‐CD8 (Biolegend). Following fixation/permeabilization (Cytofix/Cytoperm, BD Biosciences), intracellular cytokines were detected using BV421‐anti‐IFN‐γ, BV711‐anti‐TNF‐α, and APC‐anti‐IL‐2 (Biolegend). Unstimulated samples served as negative controls. Cytokine responses were derived by subtracting background signals. CEF peptide pool‐stimulated T cells were included as positive controls. Flow cytometry was performed on a BD LSRFortessa (BD Biosciences), and data were analyzed using FlowJo (BD Biosciences). Compensation was applied using single‐stained CompBeads (Biolegend) or PBMCs, while unstained PBMCs determined autofluorescence levels.

### 4.11 Quantification and Statistical Analysis

Demographic characteristics of PLWH and HD were presented as median and IQR for continuous variables and expressed as absolute values along with percentages for categorical variables. Single comparisons between two independent groups were performed using the Mann–Whitney *U* test. Multiple comparisons of antibody titers and T‐cell responses were performed using the Kruskal–Wallis test followed by a post hoc Dunn's test. NAb titer was defined as 1/4 when it was below the limit of detection. The paired NAb titers against VTT and MPXV were compared using two‐tailed Wilcoxon matched‐pairs signed‐rank test. The comparison of IgG seropositivity was performed using *Χ* ^2^ test. The VTT‐IgG titers and the frequency of VTT‐specific memory B cells were performed using the Spearman coefficient of correlation *r* ^2^. A two‐sided *p* < 0.05 was considered significant. All statistical analysis was conducted using GraphPad Prism 10.1.

## Author Contributions

J.W., R.J., L.G., and F.G. conceived and designed the study, had full access to all the data in the study, and took responsibility for the integrity of the data and the accuracy of the data analysis. R.S., Z.G., H.Z., Y.Z., L.G., Q.Z., M.F., Y.L., D.L., J.Z., and X.C. did the clinical sample collection. R.S., X.C., and N.H. did clinical data collection; M.F., Y.L., D.L., and X.C. did the clinical sample management; Q.Z., L.G., M.F., Y.L., D.L., J.Z., X.C., L.C., Y.H., and F.X. did the laboratory analysis; L.G., Q.Z., Y.P., and M.F. did the data analysis; L.G. and Q.Z. drafted the original paper; and T.D., E.A., J.W., F.G., L.G., and L.R. review and edited the paper. All authors read and edited the manuscript. All authors approved the final version, had full access to all the data, and had final responsibility for the decision to submit for publication.

## Ethics Statement

The present study was authorized by the Institutional Review Boards of Beijing Ditan Hospital Capital Medical University (2023‐025). The written informed consent was obtained from all subjects before inclusion.

## Conflicts of Interest

The authors declare no conflicts of interest.

## Supporting information




**Supporting Table 1**: Demographic characteristics of persons living with HIV (PLWH) and healthy donors in this study.
**Supporting Table 2**: The positive rate of VTT, MPXV and neutralizing antibody in people living with HIV and healthy control.
**Supporting Table 3**: The positive rate of VTT, MPXV antibodies and Cross‐reactive Memory T‐Cell Responses against MPXV in people living with HIV with different CD4 T cell counts.
**Supporting Figure 1**: Seropositivity of VTT‐IgG by year of birth in PLWH and HD born during 1949–2002.
**Supporting Figure 2**: Seropositivity of VTT‐IgG by different CD4 T‐cell counts in PLWH and HD born before 1980.
**Supporting Figure 3**: Seropositivity of MPXV‐IgG in VTT‐IgG positive individuals.
**Supporting Figure 4**: MPXV‐IgG titers in PLWH and HD presented as fold changes of area under the curve (AUC) by the year of birth.
**Supporting Figure 5**: Seropositivity of MPXV‐IgG by different CD4 T‐cell counts in PLWH and HD born before 1980.
**Supporting Figure 6**: Titers and seropositivity of VTT‐IgG MPXV‐IgG by different ART regimens in PLWH born before 1980.
**Supporting Figure 7**: Correlation between memory B cell responses and antibody titers.
**Supporting Figure 7**: Gating strategy for memory T‐cell analysis.

## Data Availability

The data of individual deidentified participants will not be shared but are available on request to the corresponding authors.
