# Supplementary material for: Impaired Cellular Immunity and Cross‐Reactive Humoral Responses to Mpox Virus in HIV‐Infected Individuals Vaccinated with Vaccinia Virus Tiantan Strain
Source: MedComm (2020). 2025 Nov 20;6(12):e70399. doi: 10.1002/mco2.70399 (PMC12635419; doi:10.1002/mco2.70399)
Supplement: Supplementary file 1 — Supporting Table 1: Demographic characteristics of persons living with HIV (PLWH) and healthy donors in this study. Supporting Table 2: The positive rate of VTT, MPXV and neutralizing antibody in people living with HIV and healthy control. Supporting Table 3: The positive rate of VTT, MPXV antibodies and Cross‐reactive Memory T‐Cell Responses against MPXV in people living with HIV with different CD4 T cell counts. Supporting Figure 1: Seropositivity of VTT‐IgG by year of birth in PLWH and HD born during 1949–2002. Supporting Figure 2: Seropositivity of VTT‐IgG by different CD4 T‐cell counts in PLWH and HD born before 1980. Supporting Figure 3: Seropositivity of MPXV‐IgG in VTT‐IgG positive individuals. Supporting Figure 4: MPXV‐IgG titers in PLWH and HD presented as fold changes of area under the curve (AUC) by the year of birth. Supporting Figure 5: Seropositivity of MPXV‐IgG by different CD4 T‐cell counts in PLWH and HD born before 1980. Supporting Figure 6: Titers and seropositivity of VTT‐IgG MPXV‐IgG by different ART regimens in PLWH born before 1980. Supporting Figure 7: Correlation between memory B cell responses and antibody titers. Supporting Figure 7: Gating strategy for memory T‐cell analysis. [file MCO2-6-e70399-s001.docx]

**Impaired Cellular Immunity and Cross-Reactive Humoral Responses to Mpox virus in HIV-Infected Individuals vaccinated with vaccinia virus Tiantan strain**

Qiao Zhang^1,#^, Rui Song^2,#^, Yu Huang^3,#^, Meiyu Fang^1, #^, Xiaoyou Chen^2,#^, Danyang Li^1^, Yanan Li^1^, Xueqi Chi^1^, Fengwen Xu^3^, Jingchuan Zhong^1^, Lan Chen^1^, Zhixia Gu^2^, Hongxin Zhao^2^, Yuanyuan Zhang^2^, Ning Han^2^, Elie Antoun^4,5^, Yanchun Peng^4,5^, Tao Dong^4,5^, Li Guo^1,6,7,*^, Fei Guo^3,6,*^, Lili Ren^1,6,7,*^, Jianwei Wang^1,4,7,*^, Ronghua Jin^2,*^

^1^NHC Key Laboratory of Systems Biology of Pathogens and Christophe Mérieux Laboratory, National Institute of Pathogen Biology, Chinese Academy of Medical Sciences & Peking Union Medical College, Beijing 100730, China.

^2^Beijing Ditan Hospital Capital Medical University, Beijing 100015, China.

^3^Center for AIDS Research, Chinese Academy of Medical Sciences & Peking Union Medical College, Beijing 100730, China.

^4^Chinese Academy of Medical Science (CAMS) Oxford Institute (COI), University of Oxford, Oxford, U.K.

^5^MRC Human Immunology Unit, MRC Weatherall Institute of Molecular Medicine, Radcliffe Department of Medicine, University of Oxford, Oxford, U.K.

^6^Key Laboratory of Pathogen Infection Prevention and Control (Ministry of Education), State Key Laboratory of Respiratory Health and Multimorbidity, National Institute of Pathogen Biology, Chinese Academy of Medical Sciences & Peking Union Medical College, Beijing 100730, China.

^7^Key Laboratory of Respiratory Disease Pathogenomics, Chinese Academy of Medical Sciences & Peking Union Medical College, Beijing 100730, China.

^#^ Equal contribution as co-first authors

^*^ Equal contribution as co-senior authors

Table S1. Demographic characteristics of persons living with HIV (PLWH) and healthy donors in this study

|  | PLWH  (1949–1980) | | | |  | PLWH  (1981–2002) |  | Matched healthy donors  (1949–1980) | | | |  | Healthy donors  (1981–2002) |
| --- | --- | --- | --- | --- | --- | --- | --- | --- | --- | --- | --- | --- | --- |
|  | Total (n=100) | 1949–1960 (n=15) | 1961–1970 (n=31) | 1971–1980 (n=54) |  | Total  (n=107) |  | Total  (n=50) | 1949–1960 (n=8) | 1961–1970 (n=15) | 1971–1980 (n=27) |  | Total (n=15) |
| **Age (year)** |  |  |  |  |  |  |  |  |  |  |  |  |  |
| Median (IQR) | 52 (47–59) | 66 (65–69) | 57 (55–60) | 47 (44–50) |  | 34 (30–38) |  | 52 (47–59) | 68 (65–69) | 58 (56–59) | 47 (45–51) |  | 34 (27–41) |
| **Sex, n (%)** |  |  |  |  |  |  |  |  |  |  |  |  |  |
| Male | 75 (75.0%) | 11 (73.3%) | 28 (90.3%) | 36 (66.7%) |  | 102 (95.3%) |  | 35 (70%) | 7 (87.5%) | 11 (73.3%) | 16 (59.3) |  | 12 (80%) |
| Female | 25 (25.0%) | 4 (26.7%) | 3 (9.7%） | 18 (33.3%) |  | 5 (4.7%) |  | 15 (30%) | 1 (12.5%) | 4 (26.7%) | 11 (40.7%) |  | 3 (20%) |
| **VTT vaccination, n (%)** |  |  |  |  |  |  |  |  |  |  |  |  |  |
| Vaccination | 100 (100%) | 15 (100%) | 31 (100%) | 54 (100%) |  | 0 (0%) |  | 50 (100%) | 8 (100%) | 15 (100%) | 27 (100%) |  | 0 (0%) |
| **Years from HIV diagnosis, n (%)** |  |  |  |  |  |  |  |  |  |  |  |  |  |
| <1 | 5 (5.0%) | 0 (0%) | 4 (12.9%) | 1 (1.9%) |  | 11 (10.3%) |  | NA | NA | NA | NA |  | NA |
| 1–3 | 8 (8.0%) | 1 (6.7%) | 4 (12.9%) | 3 (5.6%) |  | 13 (12.1%) |  | NA | NA | NA | NA |  | NA |
| >3 | 87 (87.0%) | 14 (93.3%) | 23 (74.2%) | 50 (92.6%) |  | 83 (77.6%) |  | NA | NA | NA | NA |  | NA |
| **CD4 cell count (cells/mm³), n (%)** |  |  |  |  |  |  |  |  |  |  |  |  |  |
| <350 | 13 (13.0%) | 2 (13.3%) | 5 (16.1%) | 6 (11.1%) |  | 6 (5.6%) |  | NA | NA | NA | NA |  | NA |
| 350–499 | 16 (16.0%) | 0 (0%) | 4 (12.9%) | 12 (22.2%) |  | 18 (16.8%) |  | NA | NA | NA | NA |  | NA |
| ≥500 | 68 (68.0%) | 13 (86.7%) | 20 (64.5%) | 35 (64.8%) |  | 76 (71.0) |  | NA | NA | NA | NA |  | NA |
| Not available | 3 (3.0%) | 0 (0%) | 2 (6.5%) | 1 (1.9%) |  | 7 (6.5%) |  | NA | NA | NA | NA |  | NA |
| **HIV viral load strata RNA copies per mL, n (%)** |  |  |  |  |  |  |  |  |  |  |  |  |  |
| Undetectable | 66 (66.0%) | 10 (66.7%) | 21 (67.7%) | 35 (64.8%) |  | 74 (69.2%) |  | NA | NA | NA | NA |  | NA |
| Detected but ≤1000 | 31 (31.0%) | 5 (33.3%) | 8 (25.8%) | 18 (33.3%) |  | 27 (25.2%) |  | NA | NA | NA | NA |  | NA |
| >1000 | 3 (3%) | 0 (0%) | 2 (6.5%) | 1 (1.9%) |  | 6 (5.6%) |  | NA | NA | NA | NA |  | NA |
| **Concurrent STDs, n (%)** |  |  |  |  |  |  |  |  |  |  |  |  |  |
| Syphilis | 7 (7.0%) | 0 (0%) | 2 (6.5%) | 5 (9.3%) |  | 16 (15.0%) |  | NA | NA | NA | NA |  | NA |
| Gonorrhea | 0 (0%) | 0 (0%) | 0 (0%) | 0 (0%) |  | 1 (0.9%) |  | NA | NA | NA | NA |  | NA |
| Condyloma acuminatum | 1 (1.0%) | 0 (0%) | 1 (3.2%) | 0 (0%) |  | 6 (5.6%) |  | NA | NA | NA | NA |  | NA |
| Genital herpes | 0 (0%) | 0 (0%) | 0 (0%) | 0 (0%) |  | 0 (0%) |  | NA | NA | NA | NA |  | NA |
| Other STDs | 0 (0%) | 0 (0%) | 0 (0%) | 0 (0%) |  | 0 (0%) |  | NA | NA | NA | NA |  | NA |
| None of the above | 92 (92.0%) | 15 (100%) | 28 (90.3%) | 49 (90.7%) |  | 88 (82.2%) |  | NA | NA | NA | NA |  | NA |
| **ART regimens, n (%)** |  |  |  |  |  |  |  |  |  |  |  |  |  |
| 3TC+TDF+EFV | 84 (84.0%) | 13 (86.7%) | 25 (80.6%) | 45 (83.3%) |  | 87 (81.3%) |  | NA | NA | NA | NA |  | NA |
| BIC+FTC+TAF | 16 (16.0%) | 2 (13.3%) | 6 (16.1%) | 9 (16.7%) |  | 20 (18.7%) |  | NA | NA | NA | NA |  | NA |
| **Comorbidities, n (%)** |  |  |  |  |  |  |  |  |  |  |  |  |  |
| HBV | 10 (10%) | 4 (26.7%) | 2 (6.5%) | 2 (3.7%) |  | 0 (0%) |  | NA | NA | NA | NA |  | NA |
| HCV | 0 (0%) | 0 (0%) | 0 (0%) | 0 (0%) |  | 0 (0%) |  | NA | NA | NA | NA |  | NA |
| **Vaccines, n (% )** |  |  |  |  |  |  |  |  |  |  |  |  |  |
| COVID-19 | 100 (100%) | 15 (100%) | 31 (100%) | 54 (100%) |  | 107 (%) |  | NA | NA | NA | NA |  | NA |
| Influenzas | 0 (0%) | 0 (0%) | 0 (0%) | 0 (0%) |  | 0 (0%) |  | NA | NA | NA | NA |  | NA |

STD=sexually transmitted diseases; NA=Not Applicable; 3TC+TDF+EFV=Lamivudine+ Tenofovir disoproxil + Efavirenz; BIC+FTC+TAF= Bictegravir + Emtricitabine + Tenofovir alafenamide

Table S2. The positive rate of VTT, MPXV and neutralizing antibody in people living with HIV and healthy control

|  | Years of birth | | | | |
| --- | --- | --- | --- | --- | --- |
|  | 1949-1980 | 1949-1970 | 1971-1980 | p value  (1949-1970 vs. 1971-1980) | 1981-2002 |
| **VTT-IgG** |  |  |  |  |  |
| PLWH, % (n/N) | 62 (62/100) | 74 (34/46) | 52 (28/54) | 0.023 | 1.9 (2/107) |
| HD, % (n/N) | 84 (42/50) | 87 (20/23) | 82 (22/27) | 0.6 | 0 (0/15) |
| p value | 0.0059 | 0.21 | 0.0097 |  | 0.59 |
| **MPXV-IgG** |  |  |  |  |  |
| PLWH, % (n/N) | 56 (56/100) | 78 (36/46) | 37 (20/54) | <0.0001 | 0.9 (1/107) |
| HD, % (n/N) | 82 (41/50) | 91 (21/23) | 74 (20/27) | 0.11 | 0 (0/15) |
| p value | 0.0019 | 0.18 | 0.0022 |  | 0.99 |
| **Neutralizing antibody** |  |  |  |  |  |
| PLWH, % (n/N) | 9 (9/100) | 13 (6/46) | 5.6 (3/54) | 0.002 | 0 (0/107) |
| HD, % (n/N) | 32 (16/50) | 39 (9/23) | 25.8 (7/27) | 0.32 | 0 (0/15) |
| p value | 0.0008 | 0.013 | 0.014 |  | 0.99 |

HD=Healthy donor

Table S3. The positive rate of VTT, MPXV antibodies and Cross-reactive Memory T-Cell Responses against MPXV in people living with HIV with different CD4 T cell counts

| Items | CD4 T cell count (cells/mm^3^) | | |
| --- | --- | --- | --- |
|  | ＜500  % (n/N) | ≥500  % (n/N) | p values |
| VTT-IgG seropositivity | 33.3 (7/21) | 66.7 (20/30) | 0.019 |
| MPXV-IgG seropositivity | 33.3 (7/21) | 63.3 (19/30) | 0.035 |
| VTT-specific memory B-cell responses | 47.6 (10/21) | 76.7 (23/30) | 0.033 |
| IFNγ T cell responses against MPXV CD4 peptide pool | 33.3 (7/21) | 70.0 (21/30) | 0.0096 |
| IFNγ T cell responses against MPXV CD8 peptide pool | 47.6 (10/21) | 70.0 (21/30) | 0.11 |
| At least one cytokines response against MPXV CD4 peptide pool | 85.7 (18/21) | 100 (30/30) | 0.033 |
| At least one cytokines response against MPXV CD8 peptide pool | 90.5 (19/21) | 96.7 (29/30) | 0.36 |

Figure S1. Seropositivity of VTT-IgG by year of birth in PLWH and HD born during 1949–2002


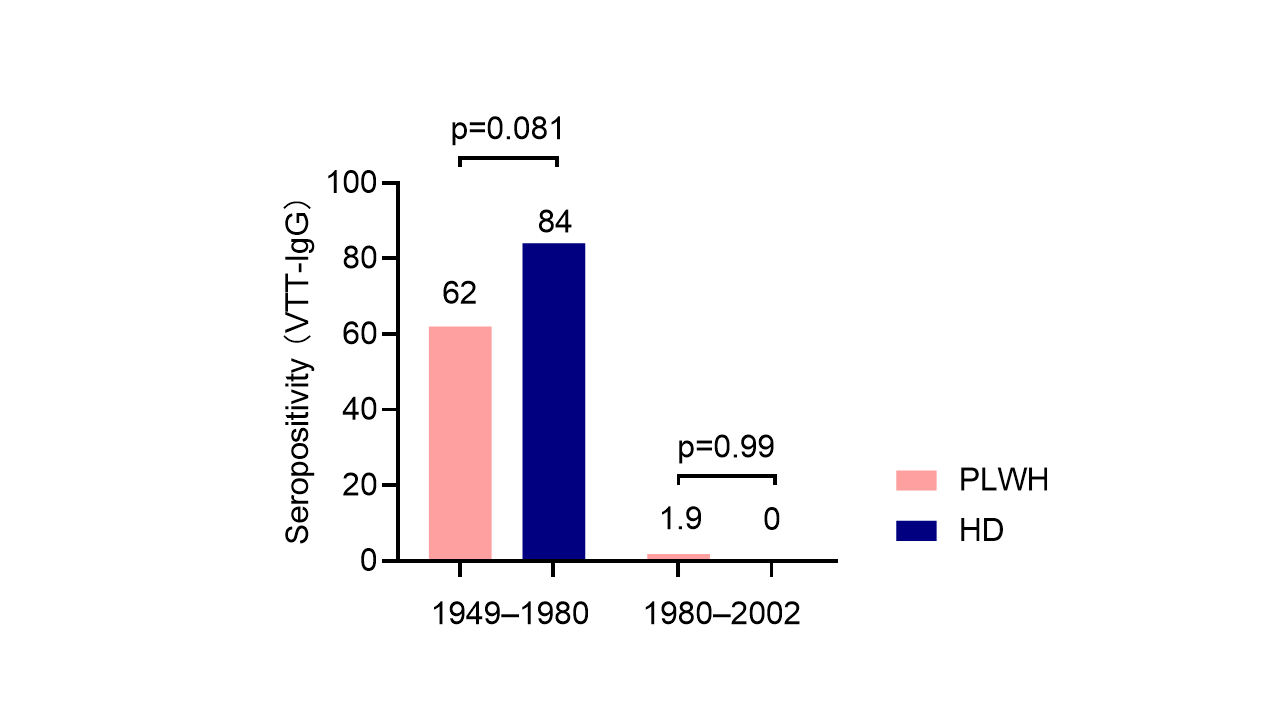


Seropositivity of VTT-IgG in PLWH and HD between individuals born between 1949–1980 (n=100 in PLWH, n=50 in HD) and those born between 1980–2002 (n=107 in PLWH, n=15 in HD). Seropositivity of VTT-IgG between PLWH and HD were compared using Chi-square test.

Figure S2. Seropositivity of VTT-IgG by different CD4 T-cell counts in PLWH and HD born before 1980.


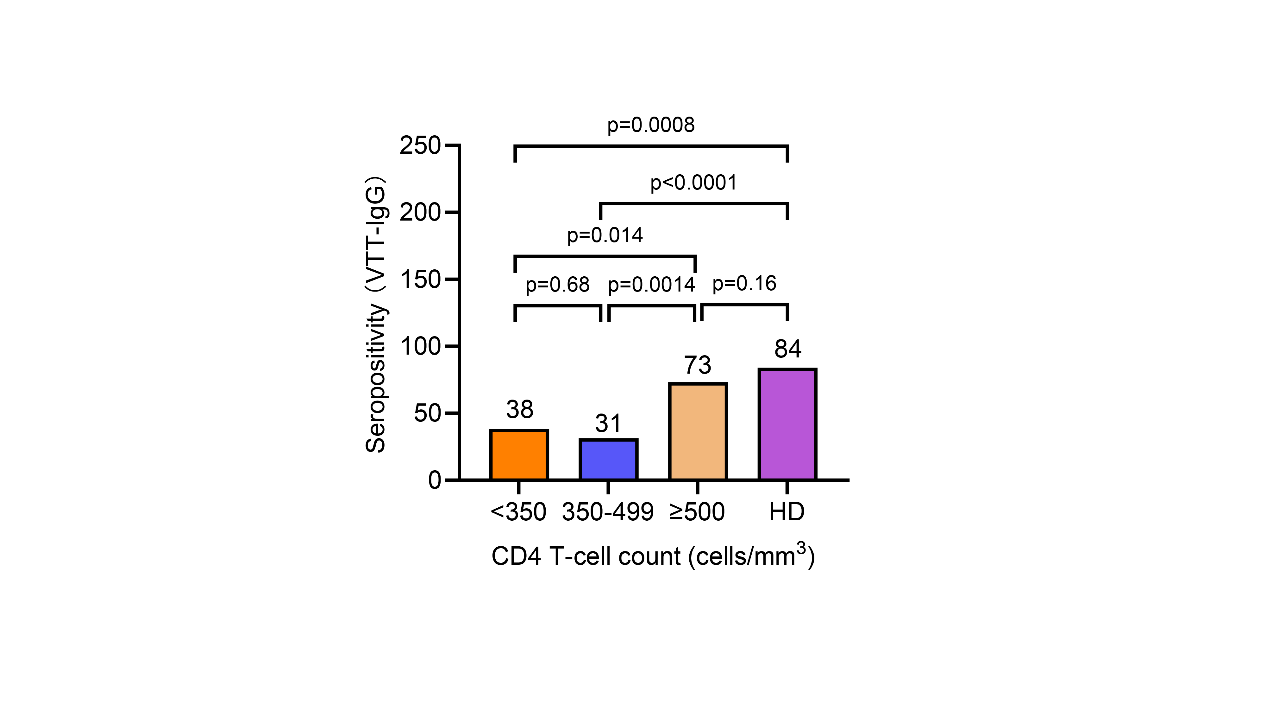


Seropositivity of VTT-IgG in HD (n=50) and PLWH (n=100) with CD4 T-cell counts less than 350 cells/mm^3^ (n=13), between 350 and 499 cells/mm^3^ (n=16) and more than 500 cells/mm^3^ (n=68). Seropositivity of VTT-IgG between HD and PLWH with different CD4 T-cell counts were compared using Chi-square test.

Figure S3. Seropositivity of MPXV-IgG in VTT-IgG positive individuals


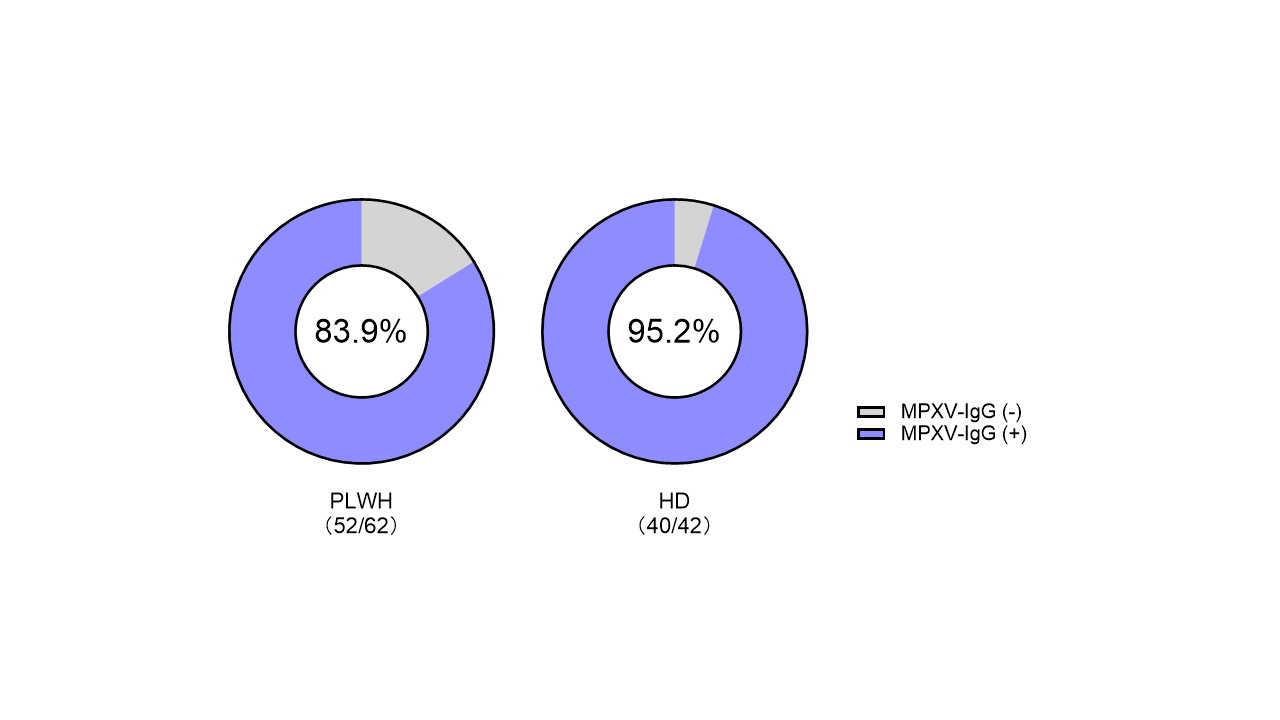


Seropositivity of MPXV-IgG in VTT-IgG positive individuals. Seropositivity of MPXV-IgG between HD and PLWH were compared using Chi-square test.

Figure S4. MPXV-IgG titers in PLWH and HD presented as fold changes of area under the curve (AUC) by the year of birth


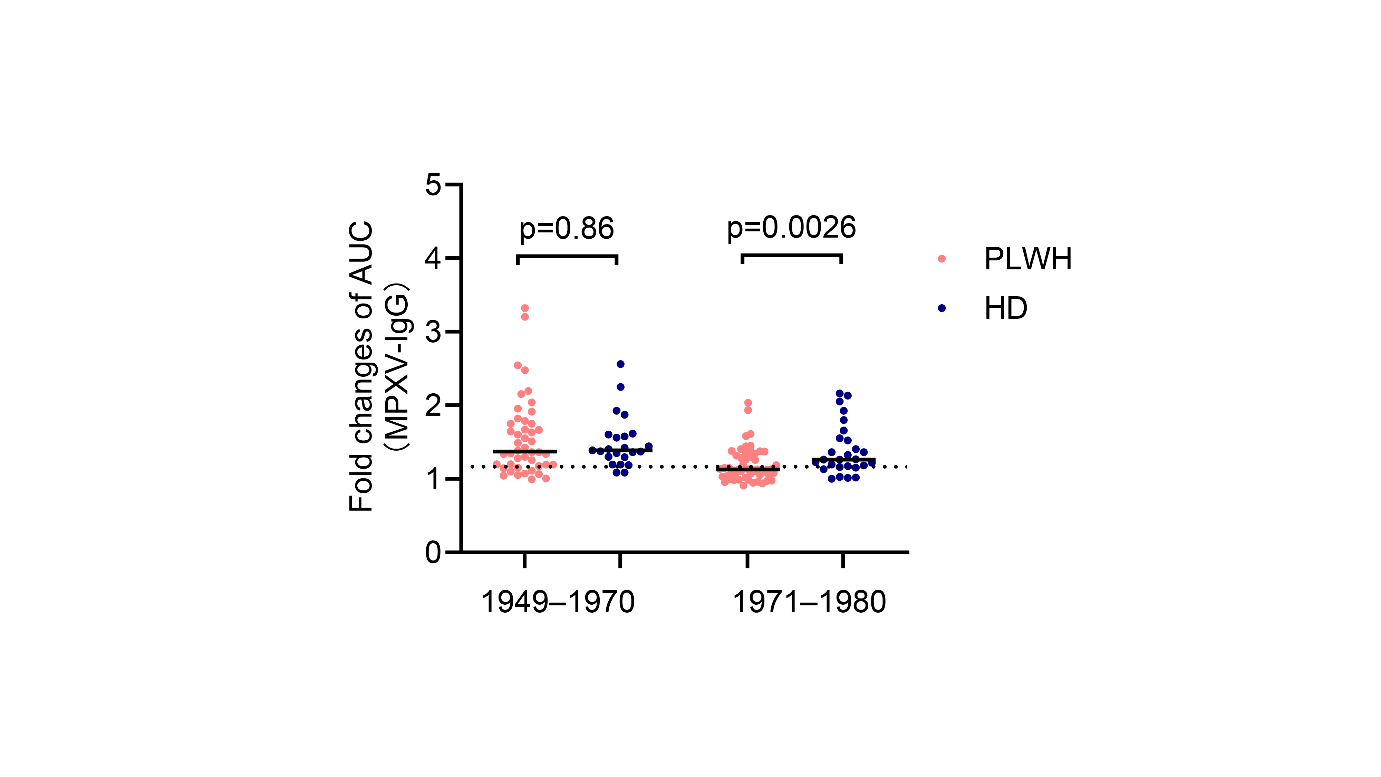


MPXV-IgG titers in PLWH and HD between individuals born between 1949–1970 (n=46 in PLWH, n=50 in HD) and those born between 1971–1980 (n=23 in PLWH, n=27 in HD). MPXV-IgG titers between PLWH and HD were compared using the Mann-Whitney U test. The dotted lines indicate the detection limit of the assays. Data are presented as Mean.

Figure S5. Seropositivity of MPXV-IgG by different CD4 T-cell counts in PLWH and HD born before 1980.


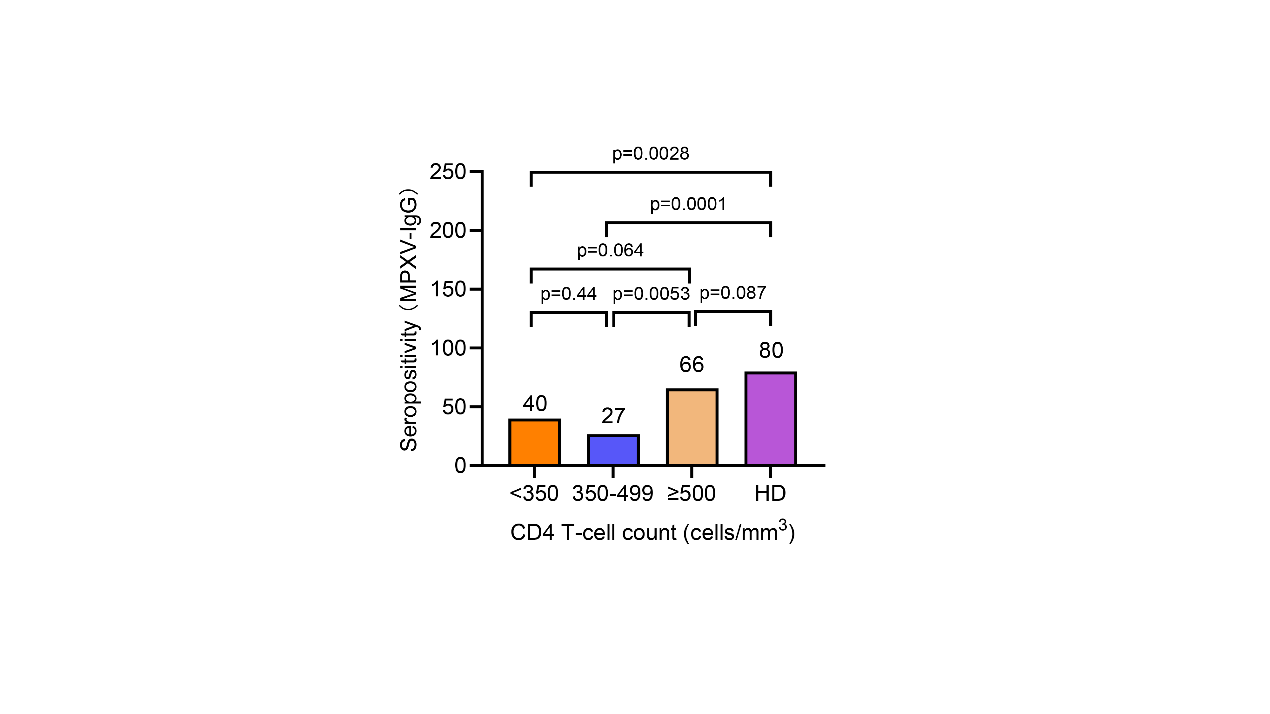


Seropositivity of MPXV-IgG in HD (n=50) and PLWH (n=100) with CD4 T-cell counts less than 350 cells/mm^3^ (n=13), between 350 and 499 cells/mm^3^ (n=16) and more than 500 cells/mm^3^ (n=68). Seropositivity of MPXV-IgG between HD and PLWH with different CD4 T-cell counts were compared using Chi-square test.

Figure S6. Titers and seropositivity of VTT-IgG MPXV-IgG by different ART regimens in PLWH born before 1980.


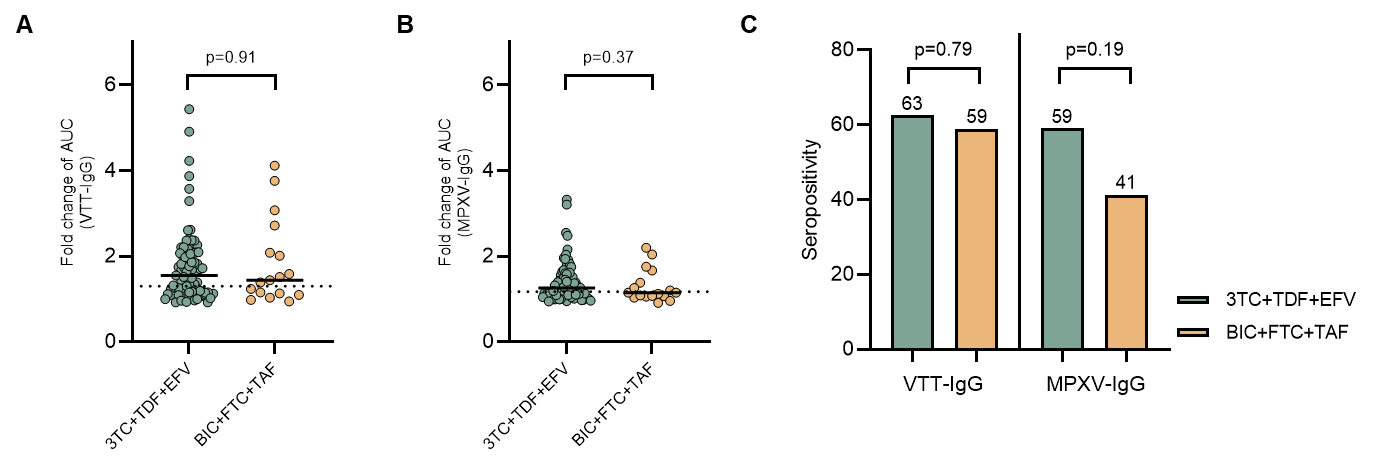


VTT-IgG titers (A) and MPXV-IgG titers (B) in PLWH (n=100) born before 1980 with ART by 3TC+TDF+EFV (n=83) and BIC+FTC+TAF (n=17). (C) Seropositivity of VTT-IgG and MPXV-IgG in PLWH (n=100) born before 1980 with ART by 3TC+TDF+EFV (n=83) and BIC+FTC+TAF (n=17). 3TC+TDF+EFV=Lamivudine+ Tenofovir disoproxil + Efavirenz; BIC+FTC+TAF= Bictegravir + Emtricitabine + Tenofovir alafenamide; IgG titers between PLWH with different ART strategies were performed by Mann-Whitney U test. Seropositivity of VTT-IgG and MPXV-IgG between PLWH with different ART strategies were compared using Chi-square test. The dotted lines indicate the detection limit of the assays. Data are presented as Mean.

Figure S7. Correlation between memory B cell responses and antibody titers.


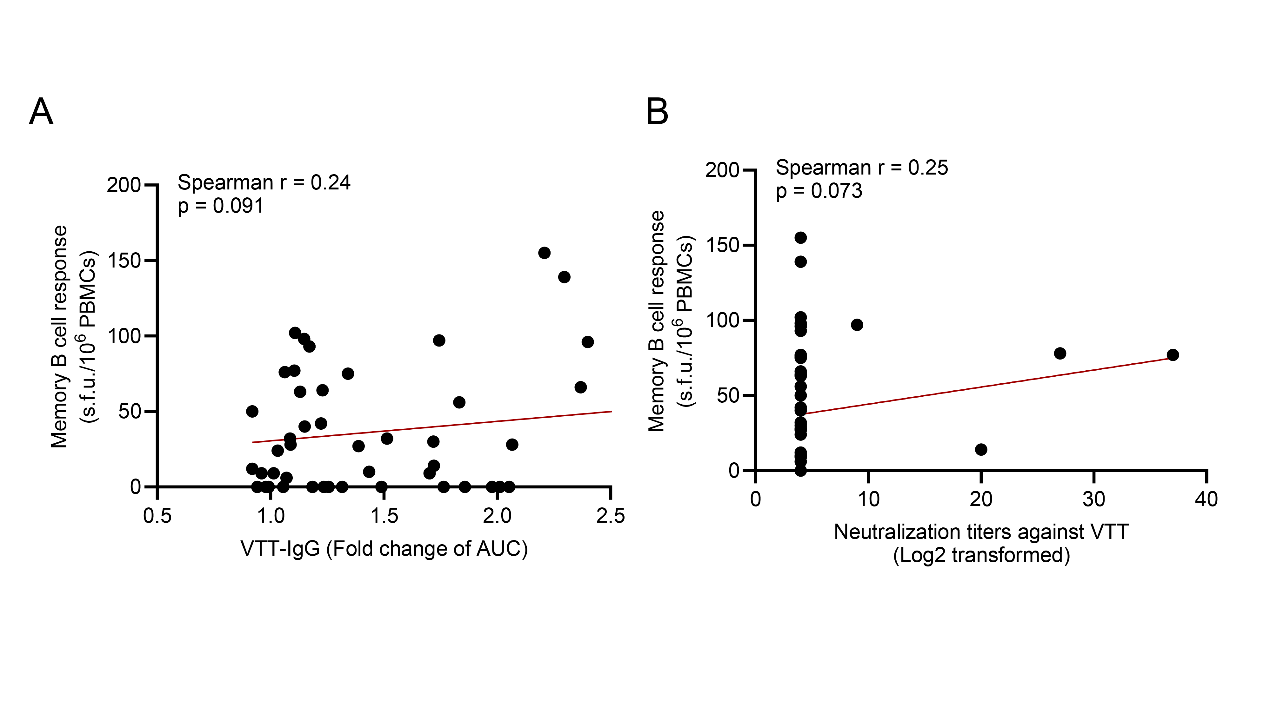
Correlation between the magnitudes of memory B cell responses and IgG (A), and neutralizing antibody (B) titers against VTT (n = 51). Spearman correlation analysis was performed.

Figure S7. Gating strategy for memory T-cell analysis


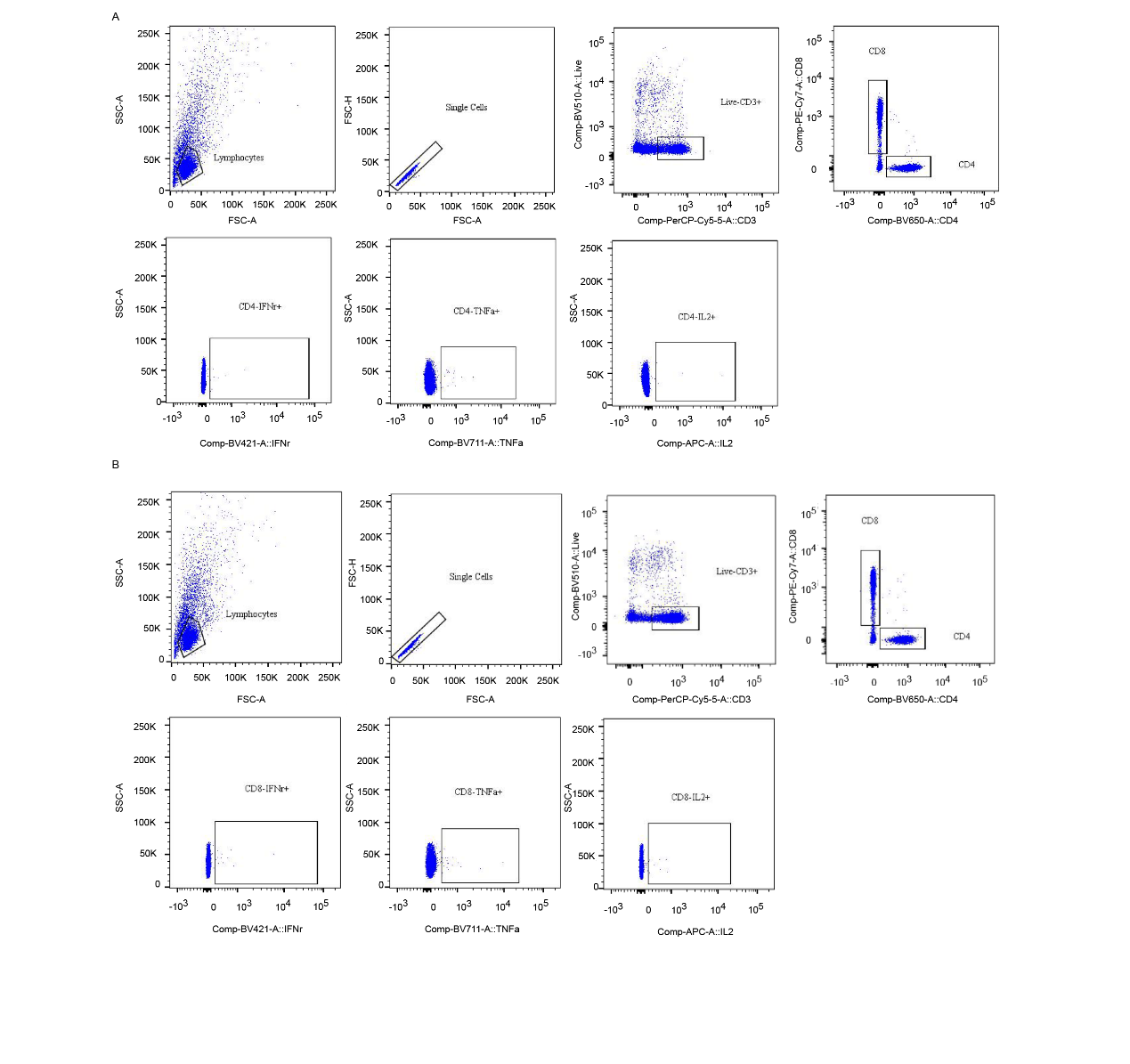


Cells were gated on single cells by a forward side scatter gate. Live T-cells were identified. After gating for CD3^+^ T-cells, and CD4^+^/CD8^+^ T-cells, IL-2+/-, IFN-γ+/-, and TNF-α+/- were gated. Population was based on corresponding negative controls.
